# Analysis of risk factors for autoimmune thyroid disease based on blood indicators and urinary iodine concentrations

**DOI:** 10.3389/fendo.2024.1453828

**Published:** 2024-11-08

**Authors:** Jianning Liu, Zhuoying Feng, Ru Gao, Peng Liu, Fangang Meng, Lijun Fan, Lixiang Liu, Yang Du

**Affiliations:** ^1^ Center for Endemic Disease Control, Chinese Center for Disease Control and Prevention, Harbin Medical University, Harbin, Heilongjiang, China; ^2^ Key Lab of Etiology and Epidemiology, Education Bureau of Heilongjiang Province & Ministry of Health (23618504), Heilongjiang Provincial Key Lab of Trace Elements and Human Health, Harbin Medical University, Harbin, Heilongjiang, China; ^3^ Department of Physical Diagnostics, Beidahuang Industry Group General Hospital, Harbin, Heilongjiang, China

**Keywords:** autoimmune thyroid disease, LASSO regression, median urinary iodine, thyroid stimulating hormone, risk factors

## Abstract

**Objective:**

The aim of this study was to elucidate the relationships between thyroid hormones, lifestyle factors, biochemical markers, and autoimmune thyroid disease (AITD), thereby identifying the factors influencing the development of these diseases.

**Methods:**

The study encompassed 517 patients with AITD and 549 patients with non-autoimmune thyroid disease. Demographic and clinical data were collected, and various laboratory indicators, including urinary iodine and thyroid hormones, were measured and compared between the groups. Lasso regression was employed to select the independent variables, while logistic regression analysis determined the factors associated with the development of AITD.

**Results:**

The prevalence of drinking alcohol history, median urinary iodine, and TSH concentrations proved significantly greater in the AITD group compared to the control group, while FT3 levels demonstrated lower values within the AITD group (p<0.05). Furthermore, there was a significant difference in the distribution of iodine nutrition status between the two groups (p<0.05). Both univariate and multivariate logistic regression analyses revealed significant associations among excessive iodine intake, drinking alcohol history, TSH, FT3, and the development of AITD.

**Conclusions:**

Excessive iodine intake and drinking alcohol history are implicated in an augmented risk of developing AITD. The prevention of AITD may necessitate the regular monitoring of TSH and FT3 concentrations.

## Introduction

Autoimmune thyroid disease (AITD) is an organ-specific autoimmune disorder characterized by the infiltration of T and B lymphocytes, as well as the production of thyroid autoantibodies (1–4). It represents one of the most prevalent health issues globally, encompassing two primary forms: Hashimoto’s thyroiditis (HT) and Graves’ disease (GD). HT is recognized as the first confirmed organ-specific autoimmune disease. In 1912, this thyroid condition was initially reported in Japan. In 1939, the term “Hashimoto’s thyroiditis” was coined by British physicians. By 1956, anti-thyroglobulin antibodies (TGAb) had been discovered in a rabbit model, and their presence in human serum was confirmed by 1958. Anti-thyroid microsomal antibodies were identified in 1959 and subsequently renamed thyroid peroxidase antibodies (TPOAb) in 1985 (1, 2, 5, 6). TPOAb has emerged as the premier serological indicator for HT, boasting a positive rate of up to 95%, and the levels of TPOAb correlate with the degree of lymphocytic infiltration within the thyroid tissue. The role of TPOAb in thyroid tissue damage remains a topic of considerable debate. The optimal diagnostic markers for HT include the presence and elevated titers of TPOAb, while ultrasonography may disclose a hypoechoic pattern in thyroid follicular structures. Fine-needle aspiration is not deemed a mandatory examination for HT. Treatment for HT is typically straightforward, often necessitating lifelong medication (7, 8). Graves’ disease (GD) represents another organ-specific autoimmune disorder, with key symptoms including anxiety, nervousness, lack of concentration, insomnia, increased appetite with weight loss, and in some patients, exophthalmos and other ocular conditions. Ultrasonography for GD typically shows diffuse thyroid hyperplasia without noticeable nodules. The primary therapeutic modalities for GD encompass antithyroid medications, radioisotope therapy, and surgical thyroidectomy (1, 7, 9). In recent years, an increase in the incidence of AITD has been observed, with varying prevalence among different demographic groups (7, 9, 10). AITD is more prevalent among women and individuals between 40 to 50 years of age (11, 12). The pathogenesis of AITD is multifaceted, with genetic and environmental factors thought to play pivotal roles in its development (3, 13). Epidemiological studies have shown that genetic factors contribute to more than 50% of the risk of AITD (14–17). In recent years, genome-wide association studies (GWAS) have identified potential associations between the genome and diseases, culminating in the discovery of several new genetic loci implicated as risk factors for AITD (18–20). Furthermore, previous epidemiological studies have identified certain environmental factors that could contribute to the pathogenesis of AITD, including iodine intake, stress, alcohol use, infectious diseases, tobacco use, and pharmacological agents (21–24). Although the pathogenesis remains incompletely elucidated, these results may offer insights to bolster the prevention of AITD and advance research into its etiology. Diagnosis of AITD primarily relies on clinical manifestations and measurement of TPOAb and TGAb levels, yet detecting AITD in its incipient stages based solely on clinical manifestations is challenging. Consequently, the development of a predictive model is imperative for the early diagnosis of AITD by amalgamating various clinical and laboratory parameters to facilitate the early detection and risk assessment of AITD.

## Materials and method

### Study subjects

From September 2023 to May 2024, this study conducted data and sample collection from inpatients and outpatients recruited at Beidahuang Industry Group General Hospital in Harbin, Heilongjiang Province, China. Inclusion criteria included: 1) individuals aged over 18 years; 2) comprehensive clinical data; 3) subjects who underwent thyroid function and biochemical level testing in the hospital’s laboratory department. Exclusion criteria comprised: 1) pregnant and lactating individuals; 2) individuals with severe hepatic or renal failure; 3) individuals with type 1 diabetes. All participants provided written informed consent, and this study received approval from the Ethics Committee of Harbin Medical University.

### Laboratory examination and diagnostic criteria

The serum levels of fasting blood glucose (FBG), triglycerides (TG), total cholesterol (TC), high-density lipoprotein (HDL) cholesterol, and low-density lipoprotein (LDL) cholesterol, were measured using the fully automated Beckman AU5800 biochemical analyzer (Beckman Coulter Experimental Systems Co., Ltd., Suzhou, China). Thyroid function parameters included thyroid stimulating hormone (TSH) [reference range: 0.3-4.5 mIU/L], free triiodothyronine (FT3) [reference range: 2.0-4.2 pg/mL], and free thyroxine (FT4) [reference range: 0.8-1.72 ng/dL]. Levels of TPOAb exceeding 16 IU/mL or TGAb exceeding 100 IU/mL are considered to be indicative of autoimmune thyroid disease. Levels of TSH, FT3, FT4, TPOAb, and TGAb were determined using a chemiluminescence immunoassay (New Industries Biomedical Engineering Co., Ltd., Shenzhen, China). Urine samples were collected from all patients and the urinary iodine concentration was determined using the Chinese health standard method for determining urinary iodine by As3^+^-Ce4^+^ catalytic spectrophotometry. The World Health Organization provides the following iodine nutrition criteria: deficient, MUI is ≤ 100 µg/L; adequate, 100 µg/L ≤ MUI ≤ 199 µg/L; more than adequate, 200 µg/L ≤ MUI ≤ 299 µg/L; excess, MUI is ≥ 300 µg/L (25, 26). Smoking was defined as the consumption of at least 3 cigarettes per day for a continuous period of 12 months, while alcohol consumption was defined as drinking more than 3 times a week for a continuous period of 12 months (27).

### Statistical analysis

Univariate analyses and binary logistic regressions were performed using SPSS 26, while the “glmnet” package in R 4.2.2 was utilized for lasso regression variable selection. The normality of the data was evaluated using the Kolmogorov-Smirnov test. Continuous variables with a normal distribution were expressed as mean ± standard deviation (SD), and comparisons between groups were conducted using independent sample t-tests. For non-normally distributed continuous variables, descriptive statistics included the median along with the 25th and 75th percentiles, and intergroup comparisons were made using the Mann-Whitney U test. Categorical variables were depicted as counts or percentages, and assessments were conducted using the chi-square test (χ^2^) or Fisher’s exact test. The least absolute shrinkage and selection operator (Lasso) regression analysis constitutes a method that is utilized for linear regression models, facilitating both shrinkage and variable selection. “Lambda.1se” is able to yield a model with excellent performance and a minimal number of independent variables. The selected independent variables were utilized to construct regression models via binary logistic regression, and odds ratios (OR) along with 95% confidence intervals (95% CI) were computed to discern the relationship to autoimmune thyroid disease. A p-value of < 0.05 was considered to indicate statistical significance.

## Results

### General information of the subjects

**Figure 1:**
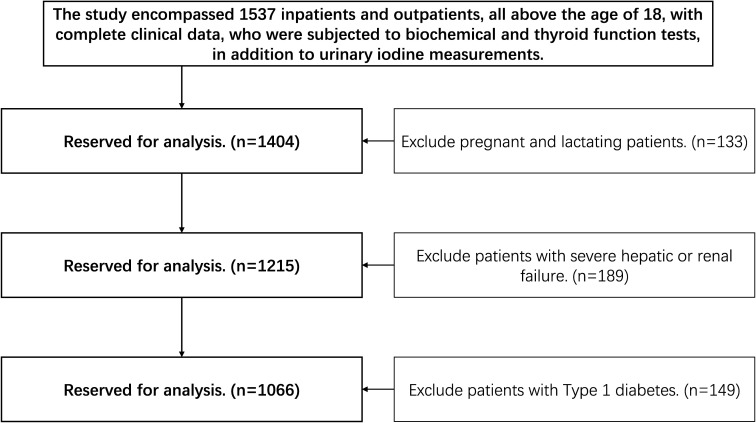
Flowchart of the participants’ screening process.

From September 2023 to May 2024, the study enrolled a total of 1537 hospitalized and ambulatory patients, each aged 18 or older and possessing complete clinical records. Participants were subjected to a series of biochemical assays, thyroid function evaluations, and urinary iodine concentration measurements. The study implemented exclusions for several groups: pregnant or breastfeeding individuals (n=133), patients with severe hepatic or renal dysfunction (n=189), and individuals diagnosed with Type 1 diabetes (n=149). The exclusion criteria yielded a final cohort of 1066 patients for analysis, comprising 517 individuals with autoimmune thyroid diseases and 549 individuals without these conditions, as illustrated in **Figure 1**.

The sex, age, smoking history, drinking alcohol history, and iodine nutrition status is detailed in **Table 1**. There were observed no statistically significant differences in sex (χ²=0.08, p=0.79), age (t=0.55, p=0.57), and smoking history (χ²=0.92, p=0.35) between the two groups. However, statistically significant differences in drinking alcohol history (χ²=14.35, p<0.01) and iodine nutrition status (χ²=66.19, p<0.01) were observed between the two groups. In the study, the proportion of individuals who consumed alcohol was observed to be significantly higher in the AITD (+) group compared to the AITD (-) group (32% vs. 22%, p<0.01). Additionally, the prevalence of adequate iodine nutrition was found to be lower in the AITD (+) group than in the AITD (-) group (16% vs. 32%, p<0.01), while the rate of excessive iodine nutrition was found to be greater in the AITD (+) group than in the AITD (-) group (41% vs. 20%, p<0.01).

**Table 1:** General information of autoimmune thyroid disease group and non-autoimmune thyroid disease group.

Variables	AITD (-)=549	AITD (+)=517	t (x^2^)	p
Age	58.31 ± 9.41	58 ± 8.74	0.55	0.57
Sex			0.08	0.79
Male	176 (32%)	170 (33%)		
Female	373 (68%)	347 (67%)		
Smoking			0.92	0.35
No	478 (87%)	460 (89%)		
Yes	71 (13%)	57 (11%)		
Drinking alcohol			14.35	<0.01
No	432 (78%)	354 (68%)		
Yes	117 (22%)	163 (32%)		
iodine nutrition status			66.19	<0.01
deficient	79 (14%)	64 (12%)		
adequate	176 (32%)	84 (16%)		
more than adequate	187 (34%)	163 (31%)		
excess	107 (20%)	206 (41%)		

AITD (-), non-autoimmune thyroid disease group; AITD (+), autoimmune thyroid disease group.

### Comparison of biochemical parameters and urinary iodine concentrations between the group with autoimmune thyroid disease and the group with non-autoimmune thyroid disease

As shown in **Table 2**, there were observed to be no statistically significant differences in FBG, TG, TC, HDL-C, and LDL-C between the two groups (p>0.05). However, the median urinary iodine of the group with autoimmune thyroid disease was found to be significantly higher than that of the group with non-autoimmune thyroid disease [257.52 (179.29, 338.37) µg/L vs. 209.69 (138.65, 273.59) µg/L, Z=-6.71, p<0.01].

**Table 2:** Comparison of urinary iodine concentrations and biochemical parameters between autoimmune thyroid disease group and non-autoimmune thyroid disease group.

Variables	AITD (-)	AITD (+)	t (Z)	p
FBG	5.94 ± 0.65	5.97 ± 0.64	-0.61	0.54
TG	2.08 ± 1.15	2.11 ± 1.17	-0.42	0.67
TC	5.31 ± 1.01	5.35 ± 0.94	-0.75	0.45
HDL-C	1.16 ± 0.31	1.14 ± 0.33	1.16	0.24
LDL-C	2.75 ± 0.85	2.82 ± 0.84	-1.21	0.23
UIC	209.69 (138.65, 273.59)	257.52 (179.29, 338.37)	-6.71	<0.01

AITD (–), non-autoimmune thyroid disease group; AITD (+), autoimmune thyroid disease group; FBG, fasting blood glucose; TG, triglycerides; TC, total cholesterol; HDL-C, high-density lipoprotein cholesterol; LDL-C, low-density lipoprotein cholesterol; UIC, urinary iodine concentrations.

### Comparison of thyroid function indicators between the group with autoimmune thyroid disease and the group with non-autoimmune thyroid disease

As shown in **Table 3**, there was observed to be no statistically significant difference in FT4 between the two groups (p>0.05). However, the median values of TSH [3.10 (1.36, 5.72) mIU/L vs. 2.63 (1.40, 3.93) mIU/L, Z=-2.98, p<0.01], FT3 [3.01 (2.70, 3.27) pg/mL vs. 3.08 (2.80, 3.29) pg/mL, Z=-2.15, p=0.031], TGAb [225.00 (81.75, 780) IU/mL vs. 15.09 (8.36, 26.85) IU/mL, Z=-23.48, p<0.01], and TPOAb [56.10 (16.35, 593.50) IU/mL vs. 2.72 (1.45, 4.78) IU/mL, Z=-22.47, p<0.01] for the group with autoimmune thyroid disease differed significantly when compared to the group with non-autoimmune thyroid disease.

**Table 3:** Comparison of thyroid function parameters between the autoimmune thyroid disease group and the non-autoimmune thyroid disease group.

Variables	AITD (-)	AITD (+)	Z	p
TSH	2.63 (1.40, 3.93)	3.10 (1.36, 5.72)	-2.98	<0.01
FT3	3.08 (2.80, 3.29)	3.01 (2.70, 3.27)	-2.15	0.031
FT4	1.27 (1.14, 1.38)	1.24 (1.11, 1.39)	-1.11	0.26
TGAb	15.09 (8.36, 26.85)	225 (81.75, 780)	-23.48	<0.01
TPOAb	2.72 (1.45, 4.78)	56.10 (16.35, 593.50)	-22.47	<0.01

AITD (-), non-autoimmune thyroid disease group; AITD (+), autoimmune thyroid disease group; TSH, thyroid stimulating hormone; FT3, free triiodothyronine; FT4, free thyroxine; TPOAb, thyroid peroxidase antibodies; TGAb, thyroglobulin antibodies.

### Comparison of thyroid function indicators and median urinary iodine between the drinking group and the non-drinking group

As shown in **Table 4**, the drinking group had a significantly higher median urinary iodine level compared to the non-drinking group [242.61 (158.86, 336.75) µg/L vs. 229.12 (147.38, 307.83) µg/L, Z=-2.333, p<0.05]. However, the median values of TSH [2.75 (1.28, 4.63) mIU/L vs. 2.80 (1.41, 4.74) mIU/L, Z=-0.162, p=0.871], FT3 [3.07 (2.78, 3.29) pg/mL vs. 3.03 (2.75, 3.27) pg/mL, Z=-0.487, p=0.627], and FT4 [1.25 (1.13, 1.37) ng/dL vs. 1.25 (1.12, 1.39) ng/dL, Z=-0.009, p=0.992] showed no significant differences between the drinking and non-drinking groups.

**Table 4:** Comparison of thyroid function parameters and median urinary iodine between the drinking group and the non-drinking group.

Variables	Drinking alcohol (-)	Drinking alcohol (+)	Z	p
TSH	2.80 (1.41, 4.74)	2.75 (1.28, 4.63)	-0.162	0.871
FT3	3.03 (2.75, 3.27)	3.07 (2.78, 3.29)	-0.487	0.627
FT4	1.25 (1.12, 1.39)	1.25 (1.13, 1.37)	-0.009	0.992
UIC	229.12 (147.38,307.83)	242.61 (158.86,336.75)	-2.333	0.02

Drinking alcohol (-), non-drinking group; Drinking alcohol (+), drinking group; TSH, thyroid stimulating hormone; FT3, free triiodothyronine; FT4, free thyroxine; UIC, urinary iodine concentrations.

### Establishment of binary logistic regression model for autoimmune thyroid disease based on lasso regression screening

**Figure 2:**
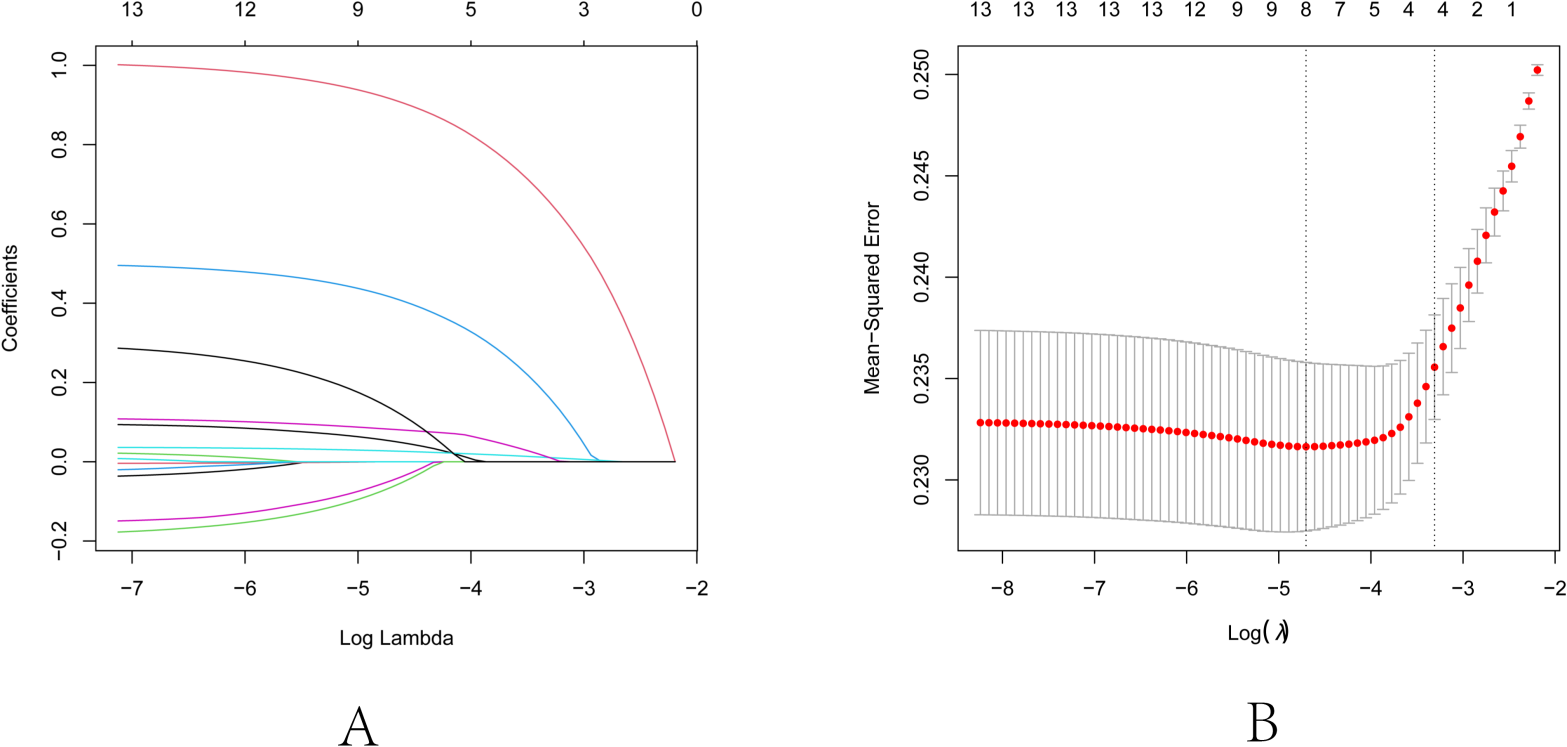
Lasso regression analysis for feature selection. **(A)** Lasso coefficient path diagram: The x-axis represents log-lambda, reflecting the degree of regularization. It shows that as the lambda value increases, the coefficient values reduce gradually to zero, and the number of retained variables decreases. **(B)** Lasso regularization path diagram: The x-axis is log (lambda); the upper axis denotes the number of non-zero coefficients, while the left y-axis represents the Mean-Squared Error (MSE). This graph demonstrates the variation in MSE with different lambda values and the confidence interval of MSE ± one standard deviation. The two vertical lines in the diagram represent the two results selected by the algorithm. The left dashed line is lambda.min, where the lambda value minimizes the MSE, and the right dashed line is lambda.1se, where the lambda value maintains the MSE within one standard error of the minimum MSE, thereby reducing the complexity of the model.

First, consolidate the categories “deficient,” “adequate,” and “more than adequate” regarding iodine status into the non-excess group. Sex, age, FBG, TG, TC, HDL-C, LDL-C, iodine excess, smoking, drinking alcohol, TSH, FT3, and FT4 were identified as independent variables, and their associations with the presence of autoimmune thyroid disease as the dependent variable were evaluated using Lasso regression. The variable selection process as the λ value evolves in the Lasso regression model is illustrated in **Figure 2**. In **Figure 2A**, the relationship between log(λ) and Lasso regression coefficient paths is illustrated, indicating that as λ increases, the degree of shrinkage in the estimated coefficients corresponding to less influential independent variables increases. This essentially leads to some coefficients being reduced to zero, consequently simplifying the model by excluding these variables. **Figure 2B** displays the curve reflecting the variable count versus log(λ). The vertical axis denotes the model’s mean square error (MSE), the lower horizontal axis signifies log(λ), and the upper horizontal axis indicates the count of non-zero coefficient independent variables at various log(λ) levels. In **Figure 2B**, the dashed line to the left indicates the optimal tuning parameter λ corresponding to the minimal MSE (lambda.min = 0.0091), while the dashed line to the right denotes the λ valued at one standard error above the minimum MSE (lambda.1se = 0.0366). For this study, the optimal model was chosen using lambda.1se = 0.0366. The analysis culminated in the selection of iodine excess, drinking alcohol, TSH, and FT3 as predictive variables, as delineated in **Table 5**. Based on the outcomes of Lasso regression analysis, logistic univariate and multivariate analyses were conducted, with autoimmune thyroid disease as the dependent variable, and iodine excess, drinking, TSH, and FT3 as the independent variables. The results, delineated in **Table 6**, demonstrate significant associations between iodine excess (OR=2.752, 95% CI: 2.078-3.643, P<0.001), drinking alcohol history (OR=1.655, 95% CI: 1.242-2.204, P<0.001), TSH (OR=1.036, 95% CI: 1.019-1.053, P<0.001), and FT3 (OR=1.174, 95% CI: 1.043-1.321, P=0.008) and the prevalence of autoimmune thyroid disease.

**Table 5:** Coefficient table of independent variables selected based on lasso regression.

Variables	Coef (lambda.min=0.0091)	Coef (lambda.1se=0.0366)
Sex	.	.
Age	.	.
FBG	.	.
TG	.	.
TC	.	.
HDL-C	-0.0103	.
LDL-C	0.0128	.
Iodine excess	0.2171	0.1614
Smoking	-0.0152	.
Drinking alcohol	0.0974	0.0394
TSH	0.0051	0.0025
FT3	0.0166	0.0027
FT4	0.0264	.

FBG, fasting blood glucose; TG, triglycerides; TC, total cholesterol; HDL-C, high-density lipoprotein cholesterol; LDL-C, low-density lipoprotein cholesterol; TSH, thyroid stimulating hormone; FT3, free triiodothyronine; FT4, free thyroxine.

**Table 6:** Binary logistic regression analysis of autoimmune thyroid disease.

Variables	Univariate		Multivariate	
	OR (95%CI)	p	OR (95%CI)	p
iodine excess	2.736 (2.078-3.602)	<0.001	2.752 (2.078-3.643)	<0.001
Drinking alcohol	1.700 (1.290-2.240)	<0.001	1.655 (1.242-2.204)	<0.001
TSH	1.03 (1.014-1.045)	<0.001	1.036 (1.019-1.053)	<0.001
FT3	1.102 (1.008-1.205)	0.033	1.174 (1.043-1.321)	0.008

TSH, thyroid stimulating hormone; FT3, free triiodothyronine.

## Discussion

AITD represents the most common autoimmune disease, characterized by immune attacks on the thyroid secondary to dysregulation of the immune system (6, 8, 28). It manifests as HT and GD, with both conditions typified by lymphocytic infiltration of the thyroid gland and elevated thyroid antibodies (29–32). Epidemiological studies indicate that the interaction between genetic susceptibility and environmental factors is pivotal to the development of AITD, although the exact mechanisms remain elusive (24, 33, 34). AITD exhibits a higher prevalence among women, a phenomenon potentially linked to estrogen levels; nonetheless, this study did not demonstrate a gender disparity in AITD prevalence.

Iodine is one of the essential trace elements for the human body and serves as a vital precursor for the synthesis of thyroid hormones, playing an important role in maintaining overall health. The physiological functions of iodine are mediated through thyroid hormones. Both iodine deficiency and excessive iodine intake can affect the morphological structure and physiological functions of the thyroid, leading to thyroid diseases (1). Research has demonstrated that excessive iodine intake among iodine-deficient populations may elevate the risk of autoimmune thyroid diseases. Furthermore, susceptible individuals with excessive iodine intake are prone to developing HT and GD, signifying a complex relationship between iodine intake levels and AITD (1, 5, 7, 9). In this study, the median urinary iodine level was found to be significantly higher in the AITD group than in the control group (257.52 vs. 209.69 μg/L, P<0.01). Within the AITD group, a higher incidence of excessive iodine nutrition was identified, indicating that iodine consumption exceeding physiological needs potentially contributes to the development of autoimmune thyroid diseases. Both univariate and multivariate logistic regression analyses revealed that excessive iodine intake constitutes a risk factor for AITD (OR=2.752, 95% CI: 2.078-3.643, P<0.001), implying that excessive iodine intake could potentially increase the risk of AITD. Following the implementation of salt iodization in China, there has been a marked enhancement in the population’s iodine nutritional status. Nonetheless, levels of circulating TGAb and TPOAb have shown an upward trend (7). The relationship between iodine intake and the levels of circulating thyroid antibodies is multifaceted. Various studies suggest that both insufficient and excessive iodine intake, compared to recommended levels, are associated with an elevation in circulating thyroid antibodies (5). Several factors contribute to the elevation of circulating thyroid antibodies subsequent to iodine fortification. First, highly iodinated thyroglobulin (Tg) can provoke an immune response against thyroid tissue owing to its potent immunogenicity. Second, elevated iodine intake upregulates the expression of intercellular adhesion molecule-1 on thyroid cells, facilitating monocyte infiltration and ensuing inflammation, a finding corroborated by mouse models of autoimmune thyroiditis (1, 22). Third, aberrant iodine consumption is linked to the anomalous expression of thyrotropin-releasing hormone-induced T helper 17 cells, suppression of regulatory T cell maturation, and dysregulated expression of TNF-related apoptosis-inducing ligand in thyroid cells, culminating in cellular apoptosis and tissue injury (1, 5). Consequently, to mitigate the risk of autoimmune thyroid disorders, it is imperative to ascertain that iodine consumption remains within the recommended thresholds. The World Health Organization stipulates a median urinary iodine concentration range of 100-200 µg/L for adults (1).

TSH, a hormone within the hypothalamic-pituitary-thyroid axis, plays a crucial role in the production of thyroid hormones (FT3 and FT4). TSH primarily regulates the proliferation of thyroid cells, blood supply to the thyroid, and the synthesis and secretion of thyroid hormones, playing a crucial regulatory role in maintaining normal thyroid function. Diseases of the pituitary gland can directly impact the synthesis and release of TSH. When abnormalities in the synthesis and secretion of thyroid hormones occur due to thyroid-related causes, they can also impact the secretion of TSH from the pituitary gland and the levels of serum TSH (35, 36). Research suggests that TSH has a significant impact on the immune system, particularly on the homeostasis of lymphocytes, suggesting its involvement in the pathogenesis of AITD (37). An increase in TSH levels might increase the risk of developing AITD (35). Inadequate iodine intake can lead to elevated levels of TSH, which may result in clinical hypothyroidism (36, 38). In this study, the median TSH level in the AITD group was substantially higher than in the control group (3.10 vs. 2.63 mIU/L, P < 0.01). Multivariate logistic regression analysis revealed a positive association between TSH levels and AITD (odds ratio (OR) = 1.036, 95% confidence interval (CI): 1.019-1.053, P < 0.001), suggesting that elevated TSH levels could increase the risk of AITD. Furthermore, the median FT3 level in the AITD group was markedly lower than in the control group (3.01 vs. 3.08 pg/mL, P = 0.031). However, multivariate regression analysis revealed a positive relationship between FT3 levels and AITD (OR = 1.174, 95% CI: 1.043-1.321, P = 0.008). It is crucial that individuals with higher TSH levels be closely monitored, and changes in FT3 levels taken into account for early prevention of AITD. Smoking and alcohol consumption have a considerable impact on the development of many diseases. However, the intricate interplay of smoking and alcohol on the immune system, as well as their potential to suppress autoimmune mechanisms, remains incompletely elucidated. The conclusions drawn from numerous studies about the relationship between lifestyle factors, including smoking history and alcohol consumption, and AITD exhibit inconsistency (3). One perspective holds that thiocyanates in tobacco exert a toxic effect on the thyroid (9). Other studies have suggested that moderate alcohol consumption may reduce the risk of developing Graves’ disease and have indicated a dose-response relationship between alcohol intake and AITD (39–42). However, the underlying mechanisms through which alcohol exposure leads to a less aggressive immune response to the thyroid remain unclear. In this study, the increased prevalence of alcohol consumption in the AITD group suggests a potential association between alcohol intake and the development of AITD. Furthermore, logistic regression analysis revealed an increased risk of developing AITD in individuals who consume alcohol (OR=1.655, 95% CI: 1.242-2.204, P<0.001). This implies that alcohol consumption could be an influential risk factor for AITD, potentially through its influence on immune system functionality and thyroid homeostasis. Meanwhile, in this study, we also analyzed the relationship between alcohol consumption, thyroid function parameters, and iodine nutrition. We found that the median urinary iodine concentration in the drinking group was significantly higher than that in the non-drinking group (242.61 vs. 229.12 μg/L, P<0.05). This phenomenon may be attributed to the higher consumption of high-iodine diets among drinkers, which suggests that drinking behavior can influence dietary habits to a certain extent. No impact of smoking on the onset of AITD was discerned in this study. Consequently, to safeguard thyroid health, the adoption of healthy habits, such as smoking cessation and moderate alcohol consumption, is crucial for mitigating the risk of AITD onset. A study indicated that individuals with AITD could possess markedly lower levels of TG, TC, and LDL-C in comparison with healthy subjects (38); however, no significant association was noted in the current study. The application of Lasso regression analysis has significantly enhanced our understanding by identifying the core predictive variables for AITD — excessive iodine, drinking alcohol history, TSH, and FT3. Lasso regression is a regression analysis technique characterized by its ability to perform variable selection and complexity control while fitting the data. It offers two primary advantages: 1) Variable Selection: By shrinking coefficients to zero, Lasso facilitates the reduction of the number of variables in the model, essentially performing feature selection, thereby enhancing the model’s simplicity and interpretability; 2) Overfitting Reduction: Through regularization, Lasso contributes to a reduction in the model’s overfitting with respect to the training data, thus bolstering the model’s predictive power for unknown data (43, 44). By penalizing the absolute size of regression coefficients, Lasso effectively eliminates variables that contribute less, thus enhancing the interpretability of the model. This approach not only confirms the risk factors suggested by univariate and multivariate analyses but also yields a more refined model for predicting the prevalence of AITD. However, this study also has some limitations. Firstly, due to the limitations of cross-sectional studies in proposing etiological hypotheses, even with rigorous statistical adjustments, causal relationships remain unproven due to potential influences from undetected biases and confounding variables. If future research includes prospective cohort studies on AITD, deeper scientific insights into the associated risk factors will be generated. Secondly, urinary iodine levels are greatly influenced by daily dietary intake, leading to fluctuations in detected urinary iodine values, which may have a certain impact on the ultimate conclusions. Lastly, considering the single-center design of this study, it is advisable to consider conducting multi-center studies to enhance the generalizability of the research findings.

In summary, our findings emphasize the importance of factoring in lifestyle influences, such as alcohol consumption and iodine intake, in the etiology of AITD. Moreover, the notable variations in the levels of TSH and FT3 highlight their potential utility as biomarkers for detecting AITD. Although our research has delineated these associations, future studies are necessary to explicate the underlying mechanisms governing the association between these lifestyle factors and thyroid autoimmunity. Furthermore, the predictive model established in this study necessitates validation in a broader and more diverse population to ascertain its applicability and inform targeted interventions for the prevention and management of AITD.

## Conclusions

We developed a binary logistic regression model using variables identified through Lasso regression. We found that high iodine intake is strongly associated with the pathogenesis of AITD and should be a primary focus of attention. Individuals exhibiting excessive iodine intake, possessing a history of alcohol consumption, and displaying elevated TSH levels require meticulous monitoring of their thyroid health to mitigate the risk of AITD development.

## Data Availability

The datasets presented in this article are not readily available because the datasets generated during the current study are not publicly available due to privacy considerations but are available from the corresponding author on reasonable request. Requests to access the datasets should be directed to hrbmudy@126.com.
